# In vitro model of distinct catabolic and inflammatory response patterns of endothelial cells to intervertebral disc cell degeneration

**DOI:** 10.1038/s41598-020-77785-6

**Published:** 2020-11-26

**Authors:** Min Ho Hwang, Hyeong-Guk Son, Joohan Kim, Hyuk Choi

**Affiliations:** 1 Department of Medical Sciences, Graduate School of Medicine, Korea University, 80, Guro-dong, Guro-gu, Seoul, 152-703 South Korea; 2 Department of Neurosurgery, Guro Hospital, College of Medicine, Korea University, Seoul, South Korea

**Keywords:** Biomedical engineering, Mechanisms of disease, Cytokines, Inflammation, Neurotrophic factors

## Abstract

To evaluate dominant cell-to-cell paracrine interactions, including those of human annulus fibrosus (AF), nucleus pulposus (NP), and endothelial cells (ECs), in the production of inflammatory mediators and catabolic enzymes, ECs was cultured in soluble factors derived from AF or NP cells (AFCM or NPCM, respectively) and vice versa. We analysed IL-6 and -8, vascular endothelial growth factor (VEGF), matrix metalloproteinase (MMP)-1 and -3, nerve growth factor (NGF)-β, and brain-derived neurotrophic factors (BDNFs) with qRT-PCR and ELISA. We implement a microfluidic platform to analyse migration properties of AF and NP cells and ECs in 3D cultures. Our results show that IL-1β-stimulated AF cells produced significantly higher levels of IL-6 and -8, VEGF, and MMP-1 than IL-1β-stimulated NP cells. However, production of IL-6 and -8, VEGF, and MMP-3 was significantly higher in NP cells than in AF cells, under the presence of ECs conditioned medium. We observed considerable migration of NP cells co-cultured with ECs through the microfluidic platform. These results suggest that AF cells may play a major role in the initial degeneration of intervertebral disc. Furthermore, it was found that interactions between NP cells and ECs may play a significant role in the development or progression of diseases.

## Introduction

Intervertebral disc (IVD) degeneration is one of the primary causes of chronic low back pain (LBP)^1,2^. Notwithstanding its high prevalence and socioeconomic burden, the IVD degeneration accompanied by LBP has not been thoroughly elucidated in terms of its aetiologies. Under normal conditions, the IVD is an avascular and aneural organ, except for the outer third of the annulus fibrosus (AF) region. However, several clinical studies have observed blood vessels within the inner IVD region in patients experiencing LBP^3–5^. This phenomenon results from several processes, including inflammatory reactions, matrix degeneration, and angiogenesis, which can be mediated by augmenting the catabolic reactions and immoderate expressions of inflammatory mediators. During this process, there are inevitable interactions between the human IVD cells and adjacent non-IVD cells, including immune cells and endothelial cells (ECs)^2^.

The IVD consists of a heterogeneous structure composed of two major distinct tissue regions: the inner gelatinous nucleus pulposus (NP) region and the surrounding outer lamellar AF. Additionally, the vascular structure, which is formed by ECs, and free nerve endings are located in the outer third of the AF regions^6,7^. The matrix homeostasis of the IVD is preserved by regulating the balance of the extracellular matrix (ECM) synthesis and degradation. However, under degenerative IVD conditions, ECM remodelling is unbalanced and shifts toward a catabolic response regulated by ECM-modifying enzymes, such as matrix metalloproteinases (MMPs)^8^.

In the first phase of IVD degeneration, immune cells such as monocytes and macrophages infiltrate the area of the lesion through the vascular structure and secrete pro-inflammatory cytokines including IL-1β^9–11^. In this inflammatory environment, AF cells stimulated by pro-inflammatory cytokines also express several catabolic enzymes, including MMPs, inflammatory mediators, and angiogenic factors, such as IL-6, IL-8, and VEGF^2,12,13^. In the second phase of the disease, these mediators promote the continuous breakdown of ECM components. This microenvironment enables the invasion of EC tubules from the outer third of the AF region into deeper IVD regions. Furthermore, ECs produce several MMPs and inflammatory mediators necessary for matrix degradation and invasion^14–16^. Clinically, the invasion of blood vessels has been observed within deeper IVD tissues. As a result, invasive EC can interact with NP cells located in inner IVD tissues. Variations in this microenvironment due to invasive ECs or cytokine-mediated inflammatory response influence the IVD cell behaviour, such as cellular mobility and migration. This is important for the progression of IVD disorders such as disc herniation. Additionally, ECs may also produce a neurogenic factor, including a β-nerve growth factor (β-NGF) and a brain-derived neurotrophic factor (BDNF). These can induce an expression of neuronal pain-associated cation channels related to pain development^4,15,17^.

**Figure 1:**
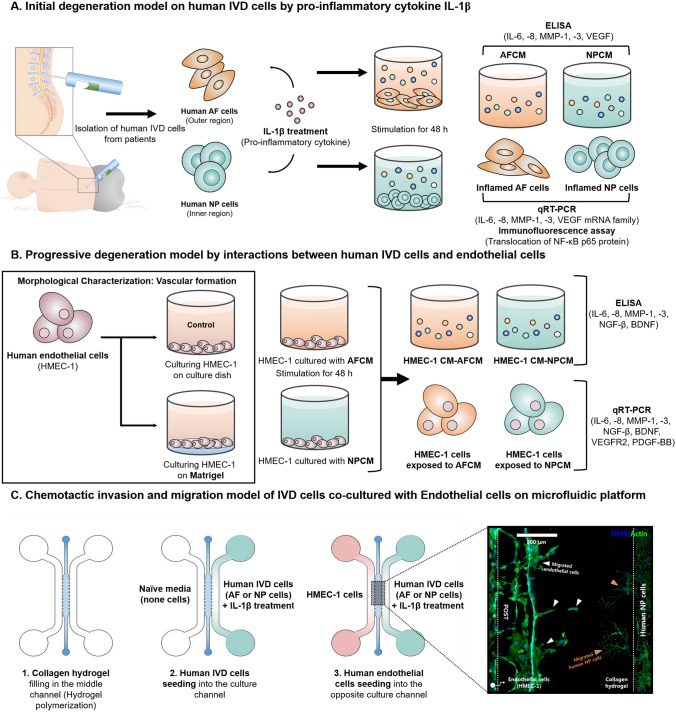
Schematic of stepwise experiment set-up used in this study. (**A**) Following initial insult, human IVD cells are stimulated by pro-inflammatory cytokines, such as IL-1β, which might be expected during initial immune cells mediated inflammatory response. Thus, we assessed the expression of inflammatory cytokines, pro-angiogenic factors, and MMPs. (**B**) In the second phase of the disease, continuous structural breakdown and excessive inflammatory response allow for the invasion of EC tubules from the outer third of the AF region into deeper IVD regions (NP region). Thus, we hypothesised that there are strong interactions between IVD cells and ECs through paracrine signalling, which might be expected during progressive IVD degeneration. (**C**) Moreover, we hypothesised that potential contributing factors due to the interactions between IVD cells and ECs can induce the chemotactic response. Thus, using microfluidic coculture devices, we verified the cellular mobility of IVD cells during these interactions, which are responsible for pain generation and disc herniation.

In this study, we hypothesised that the human AF and NP cells exhibit different responses to pro-inflammatory cytokines IL-1β or soluble factors derived from ECs, respectively With regard to the interactions between each IVD cell and the ECs, we investigated the effects of potential contributing factors, including inflammatory mediators and catabolic enzymes derived from IL-1β-stimulated IVD cells on ECs and vice versa, in order to explore the development of IVD degeneration through the secretion of inflammatory mediators and neurotrophins. Using microfluidic coculture devices, we verified the cellular mobility of IVD cells during these interactions, which are responsible for pain generation and disc herniation (Fig. 1).

## Results

### *IL-1*β *stimulation induces preferential distribution of NF-κB p65 protein into the nucleus in both human AF and NP cells*

To verify whether the NF-κB subunit p65 protein translocates into the nucleus in IL-1β-stimulated IVD cells, we analysed the preferential expression of p65 protein using the immunofluorescence method.

**Figure 2:**
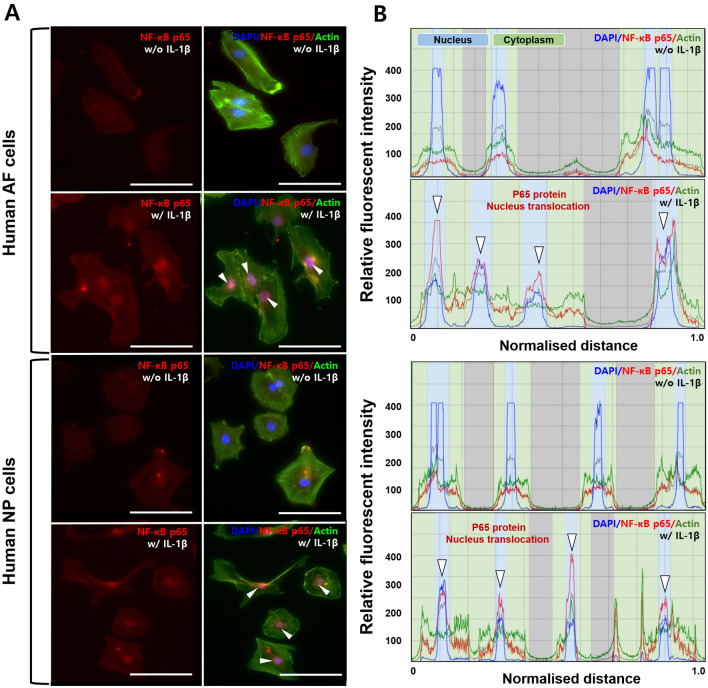
IL-1β stimulation induces nuclear translocation of NF-κB p65 protein in human AF or NP cells. (**A**) Fluorescence images of NF-κB p65 protein levels in IVD cells with/without IL-1β. (**B**) Quantification of fluorescence intensity and preferential distribution of NF-κB p65 protein levels in IVD cells with/without IL-1β. Human AF or NP cells exposed to IL-1β 10 ng/mL for 45 min revealed the translocation of p65 protein into the nucleus; this can trigger degenerative conditions because p65 protein functions as a transcription factor. Human AF and NP cells were isolated from the disc tissues of eleven patients and used at passage 2. Scale bar 100 µm.

Our fluorescence images revealed that, in both AF and NP cells, NF-κB p65 protein is located in the nucleus rather than the cytoplasm in the presence of IL-1β. This indicates the role of NF-κB p65 protein as a transcription factor (Fig. 2A). In addition, the relative intensity revealed the colocalisation of p65 protein with DAPI (Fig. 2B). These observations indicated that IL-1β stimulation can activate the inflammatory signalling pathway in both human AF and NP cells through the translocation of NF-kB p65 proteins into the nucleus.

### *IL-1*β *stimulated gene and protein expression of inflammatory mediators and ECM-modifying enzymes in human AF vs. NP cells*

To investigate the expression pattern of the inflammatory mediators and catabolic enzymes in human AF and NP cells treated with IL-1β stimulation, the gene and protein expressions of IL-6, IL-8, the VEGF family, MMP-1, and MMP-3 were measured in IL-1β-stimulated human AF or NP cells by qRT-PCR and ELISA.

**Figure 3:**
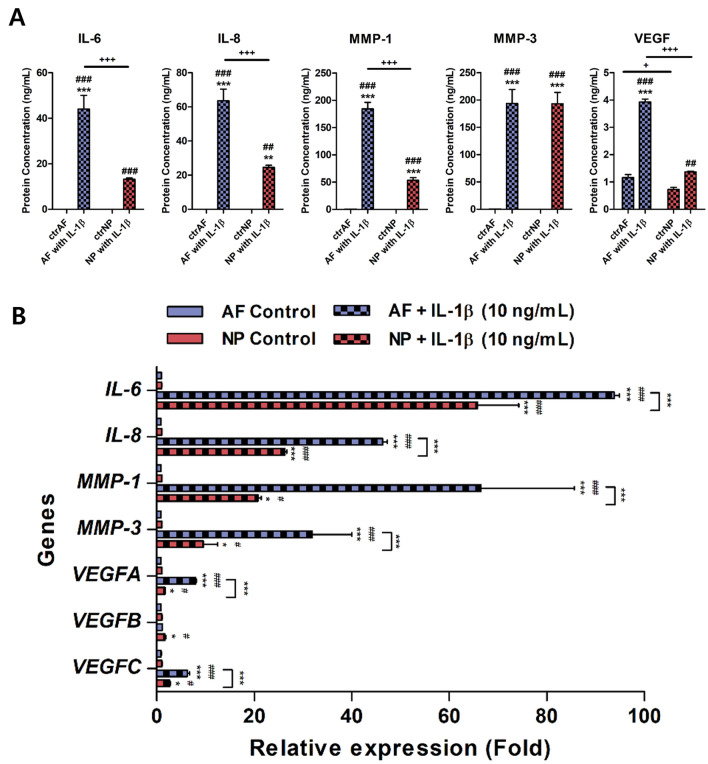
Gene and protein production of inflammatory mediators, angiogenic factor, and ECM-modifying enzymes on IL-1β-stimulated human AF *vs.* NP cells. (**A**) Protein production of inflammatory mediators IL-6, IL-8, and VEGF, and ECM-modifying enzymes MMP-1 and MMP-3 in IL-1β-stimulated human AF and NP cells. (**B**) Relative gene expression of *IL-6, IL-8, VEGF gene family (VEGFA, VEGFB, and VEGFC), MMP-1,* and *MMP-3* in IL-1β-stimulated human AF and NP cells. The values are reported as the mean ± standard error of five independent experiments. Human AF and NP cells were isolated from the disc tissues of eleven patients and used at passage 2. *p < 0.05, **p < 0.01, and ***p < 0.001 as compared with ctrAF cells. ^#^p < 0.05, ^##^p < 0.01, and ^###^p < 0.001 as compared with ctrNP cells. The line indicates comparison with each group. ctrAF, human AF cells cultured in absence of recombinant human IL-1β; ctrNP, human NP cells cultured in absence of recombinant human IL-1β; AF or NP with IL-1β, human AF or NP cells exposed to 10 ng/mL IL-1β for 48 h. After the treatment, the medium was removed and replaced with normal medium, and the cells were cultured for an additional 48 h.

The genetic and protein expressions of IL-6, IL-8, MMP-1, and MMP-3 were significantly higher in human AF and NP cells stimulated with 10 ng/mL IL-1β than in the nontreated AF and NP cells. Furthermore, there was a significantly higher expression of genetic and protein production of these factors (except for MMP-3 at the protein level) in the human AF cells than in the human NP cells (Fig. 3A,B).

With VEGF as a pro-angiogenic activator of ECs, IL-1β treatment induced the secretion of VEGF protein in both AF and NP cells. There was a significantly higher total VEGF protein levels in AF cells than in NP cells (Fig. 3A). It is noteworthy that the IL-1β stimulation induced a significantly higher expression of both *VEGFA* and *VEGFC* (which share an identical VEGF receptor, i.e., the VEGFR-2, known as the Flk-1/KDR receptor) in human AF cells than in human NP cells. However, the amount of *VEGFB* (which binds to VEGFR-1, known as the Flt-1 receptor) was higher in human NP cells than in human AF cells (Fig. 3B).

When combined, these results indicate that IL-1β stimulation induces degenerative conditions in human AF and NP cells through an abnormal production of inflammatory mediators and catabolic enzymes. Additionally, a comparison of the results for AF and NP cells indicated that AF cells function dominantly to facilitate the matrix degradation and inflammatory response under the presence of pro-inflammatory cytokine, which might be expected during the early stages of IVD degeneration.

### AFCM or NPCM modulating inflammatory mediators and ECM-modifying enzymes in HMEC-1

First, to verify that HMEC-1, used in this study as a model endothelial cells line, maintains their phenotypic feature in which capillary-like structures are formed in a culture, the cells were cultured in Matrigel for 6 h prior to the observation of their morphological alterations by using the immunofluorescence method. The cells formed capillary-like structures, and this phenotypic alteration persisted until approximately 72 h of culture (Supplementary Fig. S1). Hence, the data obtained using HMEC-1 in this study are reliable.

**Figure 4:**
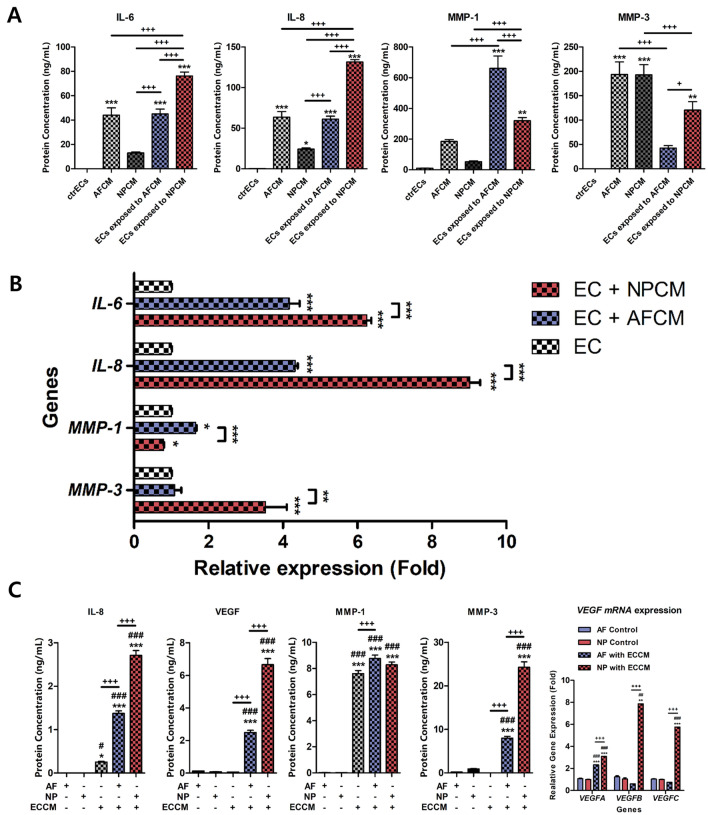
Effects of potential contributing factors derived from human AF or NP cells on HMEC-1 cells for secretion of inflammatory mediators and catabolic enzymes. (**A**) Production of IL-6, IL-8, MMP-1, and MMP-3 in HMEC-1 cells exposed to AFCM or NPCM. (**B**) Relative gene expression of *IL-6, IL-8*, *MMP-1,* and *MMP-3* in HMEC-1 cells exposed to AFCM or NPCM. (**C**) Production of IL-8, VEGF, MMP-1, MMP-3, and gene expression of VEGF mRNA family by AF or NP cells cultured in ECCM. Each value is the mean ± standard error of five independent experiments. Human AF and NP cells were isolated from the disc tissues of eleven patients and used at passage 2. *p < 0.05, **p < 0.01, and ***p < 0.001 as compared with ctrECs. The line indicates comparison with each group. ctrECs, ECs cultured in normal medium; AFCM, conditioned medium derived from human AF cells exposed to 10 ng/mL recombinant IL-1β; NPCM, conditioned medium derived from human NP cells exposed to 10 ng/mL recombinant IL-1β; ECCM, conditioned medium derived from ECs cultured in basal medium; ECs exposed to AFCM or NPCM, ECs cultured in AFCM or NPCM for 48 h. After the exposure, the medium was removed and replaced with normal medium, and the cells were cultured for an additional 48 h.

Second, to evaluate the effects of AFCM or NPCM on the production and genetic expression of IL-6, IL-8, MMP-1, and MMP-3 in HMEC-1, the cells were exposed to an AFCM or NPCM, respectively. Our results revealed that the HMEC-1 cultured in AFCM or NPCM induced a significantly higher genetic and protein production of IL-6, IL-8, MMP-1, and MMP-3, compared to when cultured in a naïve medium. Compared to human AF cells, human NP cells exhibited significantly higher genetic and protein expressions of IL-6, IL-8, and MMP-3 on HMEC-1 (Fig. 4A,B). However, HMEC-1 exposed to AFCM exhibited a higher MMP-1 production than that when exposed to NPCM.

These results indicated that human NP cells dominantly affect the gene and protein expressions of HMEC-1 during the progression of IVD degeneration. Additionally, HMEC-1 exposed to AFCM or NPCM indicated the selective induction of gene and protein expressions of MMP-1 and MMP-3 in a cell-type-dependent manner.

Conversely, both AF and NP cells cultured in ECCM exhibited significantly higher protein levels of IL-8, VEGF, MMP-1, and MMP-3, compared to those cultured in a naïve medium or only ECCM. In addition, similar to ECs, human NP cells exposed to ECCM exhibited a significantly higher expression of all the members of the VEGF family and inflammatory mediators except for MMP-1, as compared to human AF cells. However, *VEGFA* mRNA was also upregulated in human AF cells exposed to ECCM (Fig. 4C).

### Increased gene and protein expression of neurotrophic factors from HMEC-1 after culturing in AFCM or NPCM

To examine the regulation of neurotrophic factors in HMEC-1 exposed to AFCM or NPCM, we investigated the expressions of β-NGF and BDNF using qRT-PCR and ELISA. In addition, we measured the gene expressions of *VEGFR2* and *PDGF-BB*, which are known as major VEGF receptors and markers of activated ECs, respectively.

**Figure 5:**
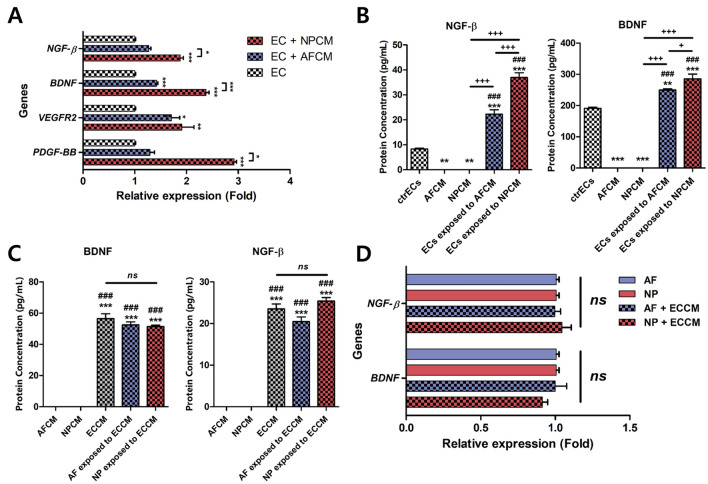
Effects of potential contributing factors derived from AF or NP cells on HMEC-1 cells for expressing neurotrophic factors and markers of activated ECs. (**A**) Relative gene expression of *VEGFR-2* (Flk-1/KDR receptor), *NGF-β*, *BDNF,* and *PDGF-BB* in HMEC-1 cells exposed to AFCM or NPCM. (**B**) Production of NGF-β and BDNF in HMEC-1 cells exposed to AFCM or NPCM. (**C**) Production of neurotrophins by AF or NP cells cultured in ECCM. (**D**) Relative gene expression of *NGF-β* and *BDNF* in AF or NP cells cultured in ECCM. Each value is the mean ± standard error of five independent experiments. Human AF and NP cells were isolated from the disc tissues of eleven patients and used at passage 2. *p < 0.05, **p < 0.01, and ***p < 0.001 as compared with the control group (ctrECs or AFCM). ^#^p < 0.05, ^##^p < 0.01, and ^###^p < 0.001 as compared with AFCM or NPCM. The line indicates comparison with each group. *Ns* no significant difference. ctrECs, ECs cultured in normal medium; AFCM, conditioned medium derived from human AF cells exposed to 10 ng/mL recombinant IL-1β; NPCM, conditioned medium derived from human NP cells exposed to 10 ng/mL recombinant IL-1β; ECCM, conditioned medium derived from ECs cultured in basal medium; AF or NP exposed to ECCM, human AF or NP cells cultured in ECCM derived from ECs cultured in normal medium.

HMEC-1 cultured in NPCM expressed significantly higher mRNA levels of *VEGFR2, PDGF-BB, NGF-β,* and *BDNF*, compared to that cultured in the naïve medium. HMEC-1 cultured in AFCM also exhibited a significant increase in the gene expressions of *VEGFR2* and *BDNF*, although the alternations in the expressions of *NGF-β* and *PDGF-BB* were not statistically significant (Fig. 5A). Both AFCM and NPCM induced the production of NGF-β and BDNF on HMEC-1, compared to the naïve medium (Fig. 5B). There were significantly higher genetic expressions of *NGF-β, BDNF,* and *PDGF-BB* in HMEC-1 exposed to NPCM as compared to those exposed to AFCM. In addition, NPCM induced a higher protein expression of NGF-β and BDNF in HMEC-1. The gene and protein expressions of NGF-β and BDNF in human AF or NP cells exposed to ECCM did not exhibit significant differences when compared to only ECCM (Fig. 5C,D). These results indicated that β-NGF and BDNF were not synthesised by human AF or NP cells. Rather, these proteins were produced exclusively by HMEC-1 cultured in AFCM or NPCM.

### Directional migration of human NP cells for interaction with HMEC-1 using microfluidic coculture platform

Based on the abovementioned results, we hypothesised that there are dominant interactions between human AF or NP cells and ECs, which are responsible for the secretion of inflammatory mediators and neurotrophins during symptomatic IVD degeneration. Thus, we assessed the migratory properties of human AF or NP cells co-cultured with HMEC-1 using our previously developed three-chamber microfluidic devices.

**Figure 6:**
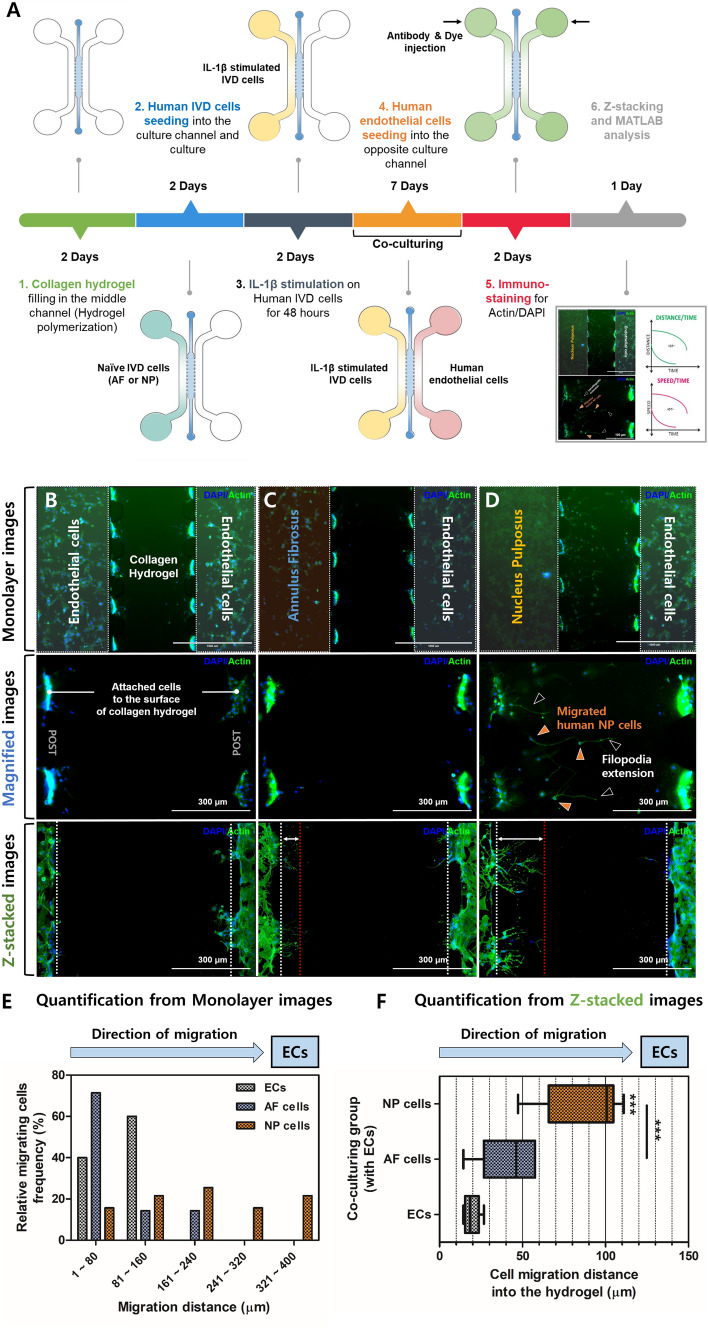
Co-culturing of human AF or NP cells with HMEC-1 cells using microfluidic coculture devices. (**A**) Schematic of microfluidic experimental design and timeline in this study. The AF or NP cells stimulated with IL-1β were plated into the left-side chamber at an approximate density of 1.0 $\times$ 10^5^ cells/mL. Thereafter, ECs were plated into the opposite-side chamber. After most ECs in the chamber were plated in a line along a sidewall of the hydrogel collagen surface, IVD and EC cells were co-cultured for seven days and maintained in a humidified atmosphere with 5% CO_2_ at 37 °C. After incubation for the designated periods, the cells were fixed and immune-stained. Fluorescence images of (**B**) HMEC-1 to HMEC-1 cells, (**C**) human AF cells to HMEC-1, and (**D**) human NP cells to HMEC-1 cultured in the microfluidic platform. Cells were three-dimensionally attached to the surface of collagen hydrogel, where they aggregated. (**E**) Quantification of migrating cell counts and migration distance from left channels toward the right channel acquired by monolayer images. (**F**) Quantification of migration and invasion distance of each cell toward the right channel acquired by Z-stacked images. The values are reported as the mean ± standard error of five independent experiments. *p < 0.05, **p < 0.01, and ***p < 0.001 as compared with the HMEC-1 to HMEC-1 culture group. The line indicates comparison with each group. Scale bar of monolayer image = 1000 µm.

Human NP cells were observed for the progressive invasion and migration into the collagen-hydrogel interface toward the right side, where potential contributing factors secreted by HMEC-1 were introduced (Fig. 6A). We also observed some directional extension of filopodia, which are the leading edge of lamellipodia in migrating cells, from human NP cells toward the HMEC-1 channel (Fig. 6B–D). Quantitative results calculated using MATLAB imaging software revealed that human NP cells co-cultured with HMEC-1 exhibited significantly higher migration distance rates and invasion into 3D collagen hydrogel than human AF cells (Fig. 6E,F).

## Discussion

The progression of IVD degeneration is characterised by two distinct yet overlapping stages. Inflammatory mediators and ECM-modifying enzymes play a major role during these phases^2^. However, there has been insufficient research regarding the dominant cell-to-cell interaction contributing to the production of these factors in each phase. Furthermore, we hypothesised that the histological locations of the inner NP cells and outer AF cells within the IVD tissues and the interactions with the adjacent non-IVD cells can provide important clues while exploring the mechanism of IVD degeneration progression.

In this study, we demonstrate that human AF cells stimulated by IL-1β, which is a major pro-inflammatory cytokine secreted by immune cells during the early stages of IVD degeneration, dominantly contribute to the genetic and protein expressions of inflammatory mediators and ECM-modifying enzymes. Meanwhile, human NP cells exposed to ECs (and vice versa) exhibit higher expressions of these factors than human AF cells. This is likely to occur during the late stages of IVD degeneration (Table 1). In addition, using the microfluidic platform, we observe the occurrence of major paracrine effects between human NP cells and ECs relevant to the progression of disc diseases through cell migration and invasion properties.

**Table 1:** Protein production of inflammatory and catabolic factors on human AF and NP cells and ECs.

Factor (protein)	IL-1β-stimulated AF cells (ng/mL)	IL-1β-stimulated NP cells (ng/mL)	Dominant response to IL-1β (group differences, p value)
IL-6	44.0433 $\pm$ 5.9945	12.2743 $\pm$ 0.2965	Human AF cells (p < 0.001)
IL-8	63.6059 $\pm$ 6.7812	24.2745 $\pm$ 1.3704	Human AF cells (p < 0.001)
VEGF	3.9264 $\pm$ 0.1075	1.3681 $\pm$ 0.0254	Human AF cells (p < 0.001)
MMP-1	184.3065 $\pm$ 11.8104	53.3675 $\pm$ 4.8277	Human AF cells (p < 0.001)
MMP-3	193.6975 $\pm$ 25.6516	193.0438 $\pm$ 20.7372	No significant difference

Factor (protein)	ECCM-stimulated AF cells (ng/mL)	ECCM-stimulated NP cells (ng/mL)	Dominant response to ECCM
IL-8	1.3738 $\pm$ 0.0576	2.711 $\pm$ 0.1099	Human NP cells (p < 0.001)
VEGF	2.4941 $\pm$ 0.1322	6.6595 $\pm$ 0.3691	Human NP cells (p < 0.001)
MMP-1	8.7663 $\pm$ 0.2533	8.2840 $\pm$ 0.2041	No significant difference
MMP-3	7.9843 $\pm$ 0.4213	24.2724 $\pm$ 1.2341	Human NP cells (p < 0.001)

Factor (protein)	AFCM-stimulated ECs (ng/mL)	NPCM-stimulated ECs (ng/mL)	Dominant response to IVD-CM
IL-6	43.0222 $\pm$ 3.3577	76.2052 $\pm$ 3.2773	Human NP cells (p < 0.001)
IL-8	51.9567 $\pm$ 3.7044	152.2767 $\pm$ 13.7929	Human NP cells (p < 0.001)
MMP-1	476.2364 $\pm$ 38.5184	266.1400 $\pm$ 9.8187	Human AF cells (p < 0.001)
MMP-3	42.5862 $\pm$ 5.0322	120.4443 $\pm$ 17.4322	Human NP cells (p < 0.05)
NGF-β (pg/mL)	22.2960 $\pm$ 1.7350	36.9920 $\pm$ 1.8440	Human NP cells (p < 0.001)
BDNF (pg/mL)	249.9617 $\pm$ 3.9420	285.8763 $\pm$ 14.8395	Human NP cells (p < 0.05)

### Dominant inflammatory response and initial IVD degeneration by human AF cells with IL-1β stimulation

In the first phase, an initiating event, such as a mechanical trauma, genetic factor, or infection, leads to phenotypic alterations in the IVD tissues. This promotes spinal instability and/or structural deficits, such as annular fissures, that are likely to result in the recruitment of immune cells into the IVD tissue^19–21^. Using this route, circulating immune cells infiltrate the deficit region and secrete a number of pro-inflammatory cytokines, such as (primarily) IL-1β^10,11^. A number of IL-1β functions, including the chemoattraction of neutrophils and catabolic response, are common to AF and NP cells^22^. These are likely to create a microenvironment favourable for angiogenesis^10,12,23,24^. The IL-1β-mediated NF-κB (p65 and p50 subunits) signal transduction pathway is well known to control the expression of a number of inflammatory and catabolic genes^2^. In our previous study and the current study, IL-1β stimulation induced the translocation of p65 protein into the nucleus, resulting in the upregulation of IL-6, IL-8, MMP-1, and MMP-3 in human AF or NP cells^25–27^.

IVD cells from degenerative conditions secreted and expressed VEGF under various experimental conditions^13,28,29^. This molecule has the potential to promote angiogenesis by acting on ECs. In mammals, the VEGF family is composed of VEGF-A, -B, -C, and -D types and a placenta growth factor. Each type of VEGF binds to different surface receptors with affinity and selectivity^30^. Currently, three major VEGF receptors, namely VEGFR-1 (known as Flt-1), -2 (KDR/Flk-1), and -3 (Flt-4), have been identified. Generally, VEGFR-1 is required for the recruitment and migration of immune cells including monocytes and macrophages. Meanwhile, VEGFR-2 and -3 have a potential role in the functions of vascular endothelial and lymph ECs. VEGF-A can bind to both VEGFR-1 and VEGFR-2, whereas VEGF-B and VEGF-C bind to VEGFR-1 and VEGFR-2, respectively^31^.

It is noteworthy that, according to our results, human AF cells exposed to IL-1β expressed higher levels of VEGF-A and VEGF-C mRNA than human NP cells, and human NP cells also expressed all the VEGF mRNA family members. These results indicate that human AF cells respond dominantly to IL-1β stimulation for an immune response. Apart from matrix degradation, IL-1β stimulation induced the upregulation of genes encoding ECM-degrading enzymes, including MMPs and a disintegrin and metalloproteinase with thrombospondin motifs (ADAMTSs), in IVD cells^8,24,32,33^. In herniated and degenerative IVD, there is a marked increase in the expression of MMPs and ADAMTSs^34^. These are responsible for angiogenesis through the enhancement of the permeability of ECs into the matrix^35^. In our results, both human AF and NP cells with IL-1β stimulation exhibited an abnormal production of MMP-1 and -3; however, the expressions in AF cells was higher than that in NP cells.

Based on these results, we infer that the human AF cells located in the outer region of the IVD are more likely to interact with and recruit circulating immune cells, as compared to the NP cells in the inner region. Hence, we conclude that the relatively higher expression of these molecules in human AF cells can provide a chemotactic path for immune cells and establish a microenvironment for the initial IVD degeneration.

### Progression of IVD degeneration and angiogenesis by dominant interaction between human NP and ECs

During the second phase, sprouting and invasive ECs in deeper NP regions can interact with human NP cells located in the inner IVD^3^. In this study, HMEC-1 cultured in AFCM or NPCM exhibited higher gene and protein expressions of IL-6, IL-8, MMP-1, and MMP-3, compared to those cultured in the naïve medium.

It is noteworthy that, unlike those under IL-1β stimulation, HMEC-1 cultured in NPCM exhibited higher levels of these expressions compared to AFCM, except for the MMP-1 levels. MMP-1 as collagenases predominantly cleaves to fibrillary collagens, particularly type 1 collagen. MMP-3 can proteolyze proteoglycans and type 2 collagen. It is generally established that the major ECM component is type 1 collagen in AF and type 2 collagen in NP^2^. Similarly, our results revealed that HMEC-1 exposed to AFCM or NPCM selectively upregulated MMP-1 and MMP-3 for both protein and gene expressions. In the second phase of the IVD disease, this shifted expression pattern of these molecules by the interaction between ECs and NP cells induce the nerve in-growth into deeper IVD region, further amplifying the inflammatory response and development of IVD disease.

ECs are primarily responsible for angiogenesis. Previous studies showed that angiogenesis is also associated with nerve in-growth. Additionally, these phenomena have been observed in painful rheumatic arthritis and osteoarthritis^14,15^. An important study showed that nerve-ingrowth into deeper NP region rather than AF region is observed in disc tissues from patient with LBP^3^. In other words, interactions between ECs and NP may play significant roles in the pathophysiology of the symptomatic disc.

### Probable mechanism of nerve innervation through invasion of ECs and secretion of neurotrophins

Newly formed vascular structures are generally associated with nerve fibre ingrowth in IVD degeneration^36^. A previous clinical study observed that in the presence of sciatica or painful conditions, nerve fibres originating from the dorsal root ganglion (DRG) are innervated within deeper NP regions and are accompanied by microvascular blood vessels^37^. A study also noted that nociceptive nerve innervation is linked to the production of β-NGF secreted by microvascular structures^4^. Other research groups showed that both β-NGF and BDNF contribute to the expression of neuronal pain-associated cation channels, such as acid-sensing ion channel 3 (ASIC3) and the transient receptor potential cation channel in the DRG. This is likely to induce irritation of nerve fibres^38–40^.

In the present study, HMEC-1 cultured in AFCM or NPCM exhibited an increased gene and protein expressions of β-NGF and BDNF. It is noteworthy that HMEC-1 exposed to NPCM expressed higher levels of these molecules than those exposed to AFCM. These results indicated that human NP cells play a major role in this molecular expression. As mentioned above, IVD tissues from patients with painful symptom showed nerve in-growth within deeper NP region. Furthermore, in degenerative IVD, much of the neovascularization is also associated with nerve innervation into NP region. Other research demonstrated that nociceptive nerve in-growth into painful discs is closely linked to NGF and BDNF production by blood vessels growing into the IVD. Together, we speculated that there is a strong interaction between NP cells and ECs^4,38–40^. In parallel, we observed that human NP cells exhibited stronger response to human ECs through migration and invasion on the microfluidic platform.

It is noted that our study has some limitations. First, because we used the IVD cells obtained from surgical patients with degenerative condition, there are the lack of “naïve” non-degenerative disc cells. Furthermore, there may be infiltrating leukocytes in the isolated AF and NP tissues/cells, which could influence the production of protein and genes on AF or NP cells used in this study.

An enhanced understanding of the contributors, including the major molecules and types of cells, in the stepwise phase of IVD degeneration could enable the identification of novel therapeutic targets and the effective treatment of symptomatic IVD disease. The application of microfluidic platforms can aid the quantification of the potency of various cytokines and chemokines that have been detected in IVD tissues (alone and in combination) and in modulating the recruitment and ingrowth mechanisms of non-IVD cells, including immune, endothelial, and neuronal cells.

## Methods

### Isolation, culture, and IL-1β stimulation of human AF/NP cells, and production of conditioned medium

Human AF and NP tissues were removed from the disc tissues of eleven patients during surgery for degenerative spinal disease (mean age ± SE = 52.18 ± 3.25; female:male = 4:7; Pfirrmann grade II–III), according to the regulations set by the institutional review board of Korea University Guro Hospital (KUGH17208-001). The AF and NP tissues were dissected through the standard procedure for IVD en bloc resection during open surgery. When extracting IVD through en bloc resection, the AF-NP junction/interface zone was removed in order to avoid heterogeneous cell mixture. Written informed consent was obtained from the patients. All the methods and experimental protocols were carried out in accordance with relevant guidelines and regulations. The cells were isolated and cultured in the nutrient mixture F-12 (Gibco-BRL) supplemented with 10% foetal bovine serum (FBS; Gibco-BRL) and 1% penicillin/streptomycin (P/S; Gibco-BRL). After two days, IVD cells were plated onto 75 cm^2^ culture flasks containing F-12 supplemented with 1% FBS and 1% P/S at a density of 5 × 10^5^ cells per flask. Human IVD cells were used at passage 2. As described above, we carried out the experiment in cell based-in vitro model. During this procedure, we could not find any infiltrated leukocytes, which may be exist in dissected IVD tissues, in the cultured AF or NP cells.

After three days, the culture medium was altered to MCDB 131 medium containing 1% P/S, 2 mM l-glutamate, and 1% FBS. This was cultured in the presence or absence of 10 ng/mL recombinant human IL-1β (R&D Systems) for an additional 48 h. After the treatment, the medium was removed and replaced with normal culture medium for an additional 48 h. In this paper, the medium collected from AF or NP cells are referred to as AF-conditioned medium (AFCM) and NP-conditioned medium (NPCM), respectively.

### Immortalised human microvascular ECs culture

HMEC-1 was cultured in MCDB 131 medium containing 10% FBS, 1% P/S, 10 ng/mL epidermal growth factor (EGF), 1 μg/mL hydrocortisone, and 2 mM L-glutamate in 75 cm^2^ culture flasks. After two days, HMEC-1 was plated and cultured in 75 cm^2^ culture flasks containing MCDB 131 medium supplemented with 1% FBS and 1% P/S at a density of 5 × 10^5^ cells per flask for 48 h. The supernatants were collected and stored at -80 °C for enzyme-linked immunosorbent assay (ELISA) analysis. In this paper, the medium collected from the HMEC-1 is referred to as the EC-conditioned medium (ECCM).

### Fabrication of microfluidic coculture platform and experimental design

We designed a microfluidic coculture platform composed of two distinct chambers connected through a hydrogel channel, using standard lithographic techniques. A patterned polydimethylsiloxane (PDMS) device was punched on two sides of the reservoirs of each chamber and bonded to a coverslip to form a closed platform. Poly-d-lysine (50 µg/mL, Sigma-Aldrich) was then deposited to enhance adhesion by preventing the detachment of the type 1 collagen hydrogel. The hydrogel solution was prepared as described in our previous study^18^. The solution was filled and gelled in the hydrogel region (without leakage) in the chamber, by means of surface tension. The medium was added to both sides of the cell culture channel, and the device was prepared for cell seeding in an incubator.

### Culturing of HMEC-1 in AFCM or NPCM

HMEC-1 was cultured with MCDB 131 medium containing 1% FBS, 1% P/S, 10 ng/mL EGF, 1 μg/mL hydrocortisone, and 2 mM L-glutamate in 75 cm^2^ culture flasks as a control group. HMEC-1 was cultured in 75 cm^2^ culture flasks with AFCM/NPCM containing 1% FBS, 1% P/S, 10 ng/mL EGF, and 2 mM L-glutamate as the experimental group for 48 h. After the treatment, the medium was removed and replaced with normal culture medium for an additional 48 h. The cells and supernatants were collected and stored at – 80 °C for qRT-PCR and ELISA analyses.

### Culturing of human AF or NP cells in ECCM

To examine the effects of the ECCM on human IVD cells, human AF or NP cells were exposed to the ECCM for 48 h. After the treatment, the medium was removed and replaced with normal culture medium for an additional 48 h. The cells and supernatants were collected and stored at – 80 °C for RT-PCR and ELISA analyses. Figure 1 shows schematics of the experimental setups used in this study.

### Immunofluorescence staining of nuclear factor kappa-light-chain-enhancer of activated B cells (NF-κB) p65 protein

To localise p65 protein expression in human AF and NP cells, the cells were plated on a glass-bottom confocal dish and exposed to IL-1β 10 ng/mL for 48 h. The disc cells were fixed with 4% paraformaldehyde and permeabilised with 0.2% Triton X-100 in PBS for 10 min at 25 °C. The cells were blocked with 5% bovine serum albumin (Millipore) in PBS and then incubated with the primary NF-κB p65 antibody (1:100; Sigma-Aldrich), followed by incubation with Alexa 555 secondary antibodies (1:200; Invitrogen). The samples were imaged using the EVOS FL auto cell imaging system (Thermo Fisher Scientific Inc., USA).

### Enzyme-linked immunosorbent assay (ELISA)

The concentrations of MMP-1, MMP-3, IL-6 and IL-8, VEGF, NGF, and BDNF in the supernatant were measured using commercially available ELISA kits (R&D Systems), according to the manufacturer’s instructions.

### Quantitative real-time polymerase chain reaction (qRT-PCR)

Human AF, NP, and HMEC-1 were lysed with TRIzol reagent (Invitrogen), RNA was extracted, and cDNA was synthesised (Life Technologies), according to the manufacturer’s instructions. qRT-PCR was performed for the mRNA of *MMP-1, MMP-3, IL-6, IL-8, VEGFA, VEGFB, VEGFC, PDGF-BB, KDR, NGF,* and *BDNF* using the SYBR Green PCR Master mix (Applied Biosystems). The mRNA expression was analysed using the 2^−∆∆Ct^ method.

### Statistical analysis

Biological samples from eleven different patients were used for five individual experiments with at least three replicates. Data were expressed as means ± 95% confidence interval (CI) for four individual experiments using independent cell cultures. One-way analysis of variance and Bonferroni’s correction post hoc test were used to assess the differences among the experimental groups. All the statistical analyses were performed using the SPSS software (version 21.3, IBM SPSS Statistics Inc., Chicago, IL, USA). A *p* value < 0.05 was considered to be statistically significant.

## Supplementary information

Supplementary Information.

## Data Availability

The datasets generated and/or analysed during the current study are available from the corresponding author upon reasonable request.
