# Supplementary material for: In vitro model of distinct catabolic and inflammatory response patterns of endothelial cells to intervertebral disc cell degeneration
Source: Sci Rep. 2020 Nov 26;10:20596. doi: 10.1038/s41598-020-77785-6 (PMC7691345; doi:10.1038/s41598-020-77785-6)
Supplement: Supplementary file 1 — Supplementary Information. [file 41598_2020_77785_MOESM1_ESM.docx]

***In vitro* model of distinct catabolic and inflammatory response patterns of endothelial cells to intervertebral disc cell degeneration**

Min Ho Hwang^1^, Hyeong-Guk Son^1^, Joohan Kim^2^ and Hyuk Choi^1,^*

^1^Department of Medical Sciences, Graduate School of Medicine, Korea University, Seoul, South Korea

^2^Department of Neurosurgery, Guro Hospital, College of Medicine, Korea University, Seoul, Korea

Reprint requests and correspondence may be sent to:

**Hyuk CHOI, PhD**

Department of Medical Sciences

Graduate School of Medicine

Korea University

80, Guro-dong, Guro-gu, Seoul 152-703, South Korea

TEL: +82-2-2626-3302

FAX: +82-2-2626-1857

E-mail: [hyuk76@korea.ac.kr](mailto:hyuk76@korea.ac.kr)


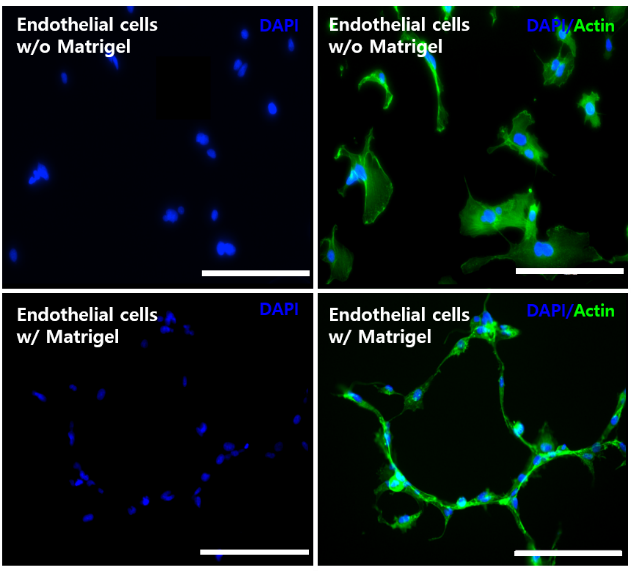


**Supplementary Figure S1. Morphologic characterization of HMEC-1**. Immunostaining images revealed that the cells had begun to form cohesive branched structures and thickened loops on the Matrigel. Scale bar = 100 µm
